# Comparison of carbon dioxide control during pressure controlled versus pressure-regulated volume controlled ventilation in children (CoCO2): protocol for a pilot digital randomised controlled trial in a quaternary paediatric intensive care unit

**DOI:** 10.1136/bmjopen-2024-087043

**Published:** 2025-01-11

**Authors:** Rebeca Mozun, Daphné Chopard, Florian Zapf, Philipp Baumann, Barbara Brotschi, Anika Adam, Vera Jaeggi, Beat Bangerter, Kristen S Gibbons, Juerg Burren, Luregn J Schlapbach

**Affiliations:** 1 Department of Intensive Care and Neonatology and Children's Research Center, University of Zurich, University Children's Hospital Zürich, Zurich, Zurich, Switzerland; 2 Department of Computer Science, ETH Zurich, Zurich, Switzerland; 3 Data Intelligence and Children's Research Center, University of Zurich, University Children's Hospital Zürich, Zurich, Zurich, Switzerland; 4 Children’s Intensive Care Research Program, Child Health Research Centre, Faculty of Medicine, The University of Queensland, Brisbane, Queensland, Australia

**Keywords:** Randomised Controlled Trial, Electronic Health Records, PAEDIATRICS, Paediatric intensive & critical care

## Abstract

**Introduction:**

Digital trials are a promising strategy to increase the evidence base for common interventions and may convey considerable efficiency benefits in trial conduct. Although paediatric intensive care units (PICUs) are rich in routine electronic data, highly pragmatic digital trials in this field remain scarce. There are unmet evidence needs for optimal mechanical ventilation modes in paediatric intensive care. We aim to test the feasibility of a digital PICU trial comparing two modes of invasive mechanical ventilation using carbon dioxide (CO_2_) control as the outcome measure.

**Methods and analysis:**

Single-centre, open-labelled, randomised controlled pilot trial with two parallel treatment arms comparing pressure control versus pressure-regulated volume control. Patients are eligible if aged <18 years, weighing >2 kg, have an arterial line and require >60 min of mechanical ventilation during PICU hospitalisation at the University Children’s Hospital Zurich. Exclusion criteria include cardiac shunt lesions, pulmonary hypertension under treatment and intracranial hypertension. CO_2_ is measured using three methods: end-tidal (continuous), transcutaneous (continuous) and blood gas analyses (intermittent). Baseline, intervention and outcome data are collected electronically from the patients’ routine electronic health records. The primary feasibility outcome is adherence to the assigned ventilation mode, while the primary physiological outcome is the proportion of time spent within the target range of CO_2_ (end-tidal, normocarbia defined as CO_2_ ≥ 4.5 and ≤ 6 kPa). Both primary outcomes are captured digitally every minute from randomisation until censoring (at 48 hours after randomisation, extubation, discharge or death, whichever comes first). Analysis will occur on an intention-to-treat basis. We aim to enrol 60 patients in total. Recruitment started in January 2024 and continued for 9 months.

**Ethics and dissemination:**

This study received ethical approval from the Cantonal Ethics Commission of Zurich (identification number: 2022–00829). Study results will be disseminated through publication in a peer-reviewed journal and other media like podcasts.

**Trial registration number:**

NCT05843123.

**STRENGTHS AND LIMITATIONS OF THIS STUDY**

- This study compares two commonly used modes of invasive mechanical ventilation in a randomised design, providing feasibility data to inform the conduct of digital trials by using electronic patient data directly extracted from the source systems, minimising manual data collection and associated bias and thereby increasing local readiness for more efficient clinical trial conduct.
- The outcomes of this pilot trial focus on feasibility and physiological measures, with the sample size allowing the assessment of feasibility and exploration of physiological findings, to guide the design and inform power calculations of future larger trials, which should also investigate patient-centred outcomes, age-related differences and longer follow-up time.
- Blinding is not possible due to the nature of the intervention.
- Technical issues that may affect the availability or accuracy of data may arise and will be documented.
- Some aspects of digital trials, such as electronic informed consent, are not implemented in this trial.

## Introduction

 Trials using digital recordings of routine clinical data have great potential to decrease the resources required to conduct clinical trials, which remain one of the major obstacles to faster evidence generation in clinical care. The coronavirus disease pandemic has demonstrated the need for rapid implementation of clinical trials in intensive care, particularly including platform or adaptive designs.^1 2^ Advantages of digital clinical trials include facilitation of recruitment, optimisation of prospective data collection, workflows and monitoring, as well as innovative methods to automate processes, make predictions or assist in phenotyping and result interpretation.^3^ Intensive care units represent an attractive setting for digital trials given the rich data environment with high temporal resolution.^4^

Approximately one in three children receives invasive mechanical ventilation when admitted to paediatric intensive care units (PICUs).^5^ Invasive mechanical ventilation can save lives while bearing risks of ventilator-induced lung injury.^6^ Other negative aspects directly related to invasive mechanical ventilation include ventilator-associated events such as ventilator-associated pneumonia and pneumothorax, side effects of sedation such as neurotoxicity, withdrawal and delirium, and family burden due to disruptive interactions. Mechanical ventilation’s main goal is to provide appropriate gas exchange. Suboptimal mechanical ventilation may lead to fluctuations in carbon dioxide (CO_2_) with associated changes in cerebral and pulmonary blood flow.^7^ These changes can aggravate cerebral ischaemia or swelling and pulmonary arterial hypertension. Prolonged intubation and delayed weaning increase the risk of chronic lung disease, prolonged PICU stay, critical illness neuromyopathy and worse patient-centred outcomes.^8^

It is not known which ventilation mode is most effective with the least harm in children, despite recent technological advances in paediatric mechanical ventilation and the availability of digital ventilation monitoring data.^9^ Classical ventilation modes include conventional pressure-controlled (PC) and volume-controlled modes. Modern ventilators offer algorithm-based adaptive ventilation modes to maximise lung protection.^10^ Pressure-regulated volume control (PRVC) is one such adaptive mode that aims to achieve optimal oxygenation and lung recruitment at lower mean airway pressures (MAP) while maintaining a defined tidal volume.^7^ These adaptive modes rapidly adjust to changes in lung compliance, thereby reducing lung injury, providing a stable minute volume, and stabilising gas exchange. In a Cochrane review, neonates (mostly preterm or low birth weight) ventilated with PRVC modes had lower rates of death or bronchopulmonary dysplasia, pneumothorax, hypocarbia, severe cranial ultrasound pathologies and duration of ventilation compared with infants ventilated with PC modes.^11^ However, there is insufficient evidence to determine the best ventilation mode in critically ill children, and clinicians’ choice is often based on personal or institutional preference.^9 12^

### Aims and hypothesis

The aims of this study are to test the feasibility of a digital trial in the PICU setting, comparing two modes of invasive mechanical ventilation, PC versus PRVC, using CO_2_ levels as the outcome measure. We hypothesise that a digital interventional trial comparing two modes of ventilation in the PICU is feasible and that children will spend more time in normocarbia when ventilated using the PRVC mode compared with the PC mode.

## Methods

### Study design and setting

This study is a pilot, interventional, single-centre, open-label, randomised controlled clinical trial to assess the feasibility of a digital PICU trial. The trial is conducted in the PICU of the University Children’s Hospital Zurich, Switzerland, and is overseen by the Children’s Research Centre monitor. This trial serves to evaluate feasibility, test procedures and obtain parameter estimates that will inform the design and the sample size of future trials. The study was initially planned to begin recruitment in June 2023 and expected to conclude within 6 months by December 2023, whereas actual recruitment started in January 2024 and was expected to be completed in July 2024. This protocol manuscript was submitted on 29 March 2024. Study recruitment was completed during the peer-review process; however, no changes were made to the protocol. We follow Standard Protocol Items for Randomised Trials reporting guidelines.^13^

### Participants, screening and consent procedures

Mechanically ventilated children who are younger than 18 years are eligible for randomisation if their weight is above 2 kg, and if they have an arterial line. Patients who have been ventilated for longer than 24 hours, and those with cyanotic heart disease, pulmonary hypertension or at risk for increased intracranial pressure are excluded. Detailed inclusion and exclusion criteria are listed in table 1. Mechanically ventilated children are screened daily from Monday to Friday by study coordinators using the institutional PICU patient data monitoring systems (MetaVision Suite, Israel-iMDsoft Ltd., Tel Aviv, Israel/CGM Clinical—Phoenix, CompuGroup Medical Switzerland AG, Niederwangen, Switzerland). If eligible, a study coordinator and a physician contact the child’s parents or legal guardian(s) to explain the study and obtain prospective consent. Written informed consent is sought from parents or guardians and adolescent patients aged 14 years and older. If prospective consent cannot be obtained for an otherwise eligible child due to the emergency environment, study procedures are initiated if approved by an independent physician and consent is sought as soon as possible once the parent/guardian has had time to adapt to the emergency environment in the PICU (consent to continue, to be sought within 24 hours). No intervention or outcome data will be available for analysis from those where consent cannot be obtained. Withdrawal of consent does not affect subsequent medical assistance and treatment of the child. In case of withdrawal of informed consent, there is no further data collection. All data collected up to that point may be analysed in coded form and after the evaluation anonymised or deleted. Patients may be enrolled more than once during subsequent PICU admissions if the date of the second enrolment is at least 30 days after the date of the previous enrolment.

**Table 1:** Inclusion and exclusion criteria

Inclusion criteria	Exclusion criteria
Admission to PICU at the University Children’s Hospital Zurich Mechanical ventilation required for >60 min during PICU hospitalisation Arterial line required during PICU hospitalisation Age <18 years Weight >2 kg Informed consent provided by the participant or the participant’s parents or legal guardians, or in case of an emergency situation approval by a physician who is independent of the study	Substantial air leak around the endotracheal tube (>30%) Cyanotic heart disease Intracranial hypertension (ie, traumatic brain injury or patients admitted after neurosurgery) Pulmonary hypertension under treatment (ie, sildenafil or inhaled nitric oxide) Time from start of invasive mechanical ventilation until screening time >24 hours Previous enrolment in the trial in the past 30 days Inability of the parents or legal guardians to understand the study due to linguistic or cognitive reasons

Need for mechanical ventilation and need for an arterial line will be based on clinical decision of the treating physician. Previous enrolment refers to previous admission. PICU refers to the paediatric intensive care unit at the University Children’s Hospital Zurich.

### Randomisation and blinding

The randomisation schedule was computer-generated with variable block sizes (4, 6, 8 and 10) using a 1:1 allocation ratio and stratified by age as follows:

- Neonates, defined as having a postnatal age <28 days if born at term (ie, born at ≥37 weeks of gestational age) or a postnatal age of <44 weeks of corrected gestational age if born preterm (ie, born at<37 weeks of gestational age).
- Older infants, children or adolescents, defined as a postnatal age ≥28 days if born at term or ≥44 weeks corrected gestational age if born preterm.

**Figure 1:**
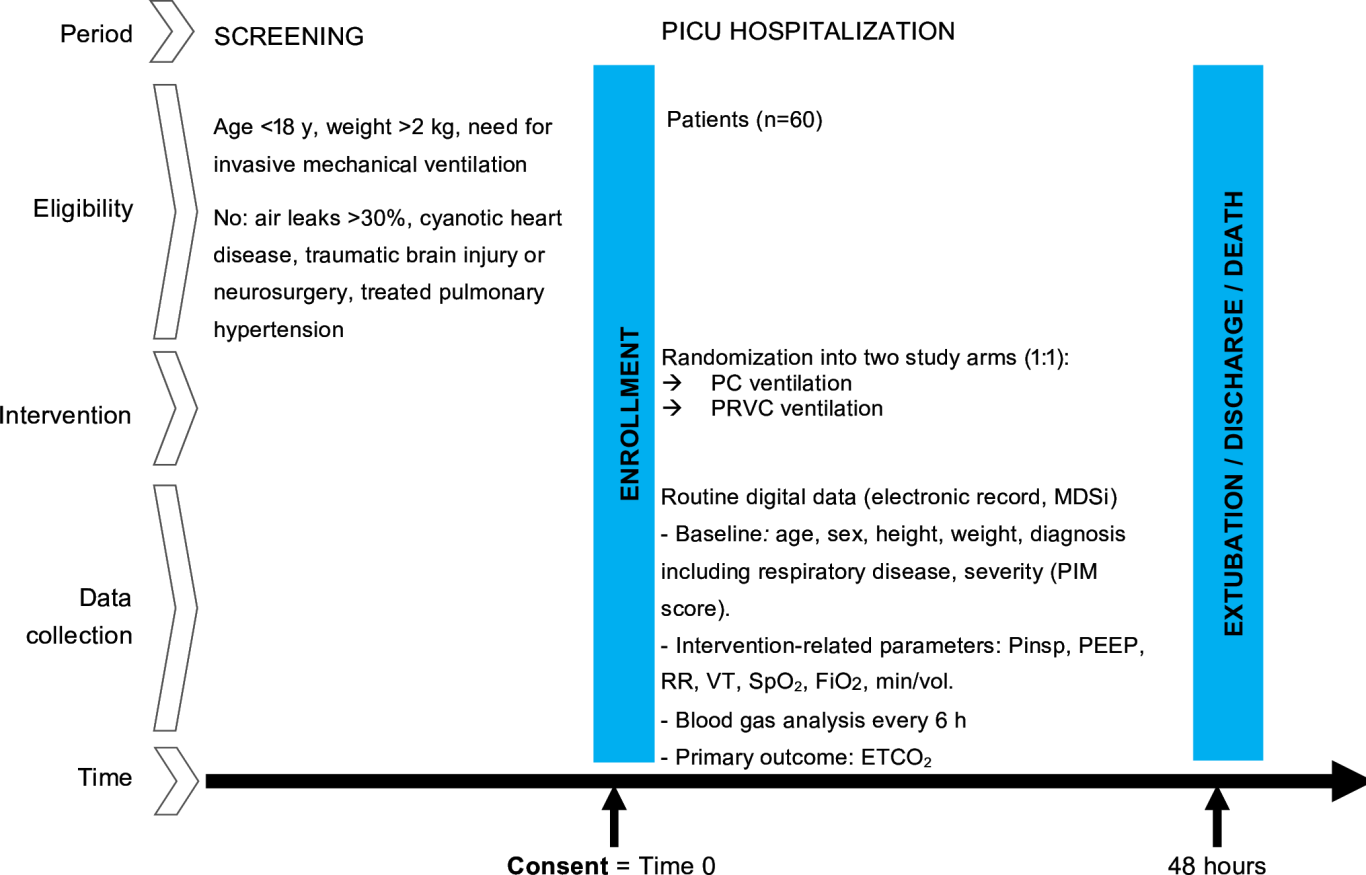
Study design of the CoCO_2_ randomised controlled trial. COCO_2_, comparison of carbon dioxide control during pressure controlled versus pressure regulated volume controlled ventilation in children; ETCO_2_, end-tidal carbon dioxide; FiO_2_, Fraction of inhaled oxygen; MDSi, minimal dataset of the Swiss Society of Intensive Care; MV, minute vol; PC, pressure control; PEEP, positive end-expiratory pressure; PICU, Paediatric intensive care unit; PIM-2, Paediatric Index of Mortality version 2; Pinsp, peak inspiratory pressure; PRVC, pressure-regulated volume control; RR, respiratory rate; SpO_2_, peripheral oxygen saturation; VT, tidal volume.

The randomisation and electronic allocation system is embedded in the trial’s Research Electronic Data Capture (REDCap) database,^14^ which ensures concealment of allocation. The randomisation sequence was generated by the data manager, who is not involved in recruitment or allocation. Based on our team’s previous experience using REDCap randomisation, we do not anticipate any challenges, and as such, plus the digital focus of the study, we do not have backup envelopes. Once eligibility is confirmed and consent is obtained from the patient, family or an independent physician, randomisation is performed by a study coordinator or physician and allocation is communicated electronically to the treating staff. Blinding is not possible due to the nature of the intervention. Eligible patients are enrolled in the trial after obtaining consent (time point 0), or consent to continue (figure 1) and randomisation.

### Interventions and treatment arms

The trial compares two ventilation modes: PC and PRVC. Both modes of invasive mechanical ventilation are part of routine standard care at the study hospital. Invasive ventilation is provided using the Bellavista 1000 ventilators (IMT Medical, Buchs, Switzerland/Vyaire Medical, Chicago, USA). The Bellavista brand label for the PC mode is ‘pressure support ventilation (PSV)’ and for the PRVC mode is ‘pressure support ventilation with TargetVent (PSV with TargetVent)’. Both ventilation modes provide pressure using a decelerating flow pattern. The difference between the modes is that in PC physicians set the inspiratory pressure (plus inspiratory time, respiratory rate and positive end-expiratory pressure (PEEP)), whereas in PRVC physicians set a target tidal volume (plus inspiratory time, respiratory rate, PEEP, as well as the upper and lower limits of pressure support), and an algorithm delivers pressure to achieve the target tidal volume based on lung compliance measured during previous breaths while remaining between the prescribed pressure limits. Deviations from the assigned mode are allowed based on the clinical judgement of the treating physician and will be captured. Measures to improve adherence include verbal reminders and placement of stickers indicating the assigned ventilation mode on the ventilation monitors.

### Study outcomes

This pilot study is designed to provide estimates of feasibility and physiological outcomes with high temporal resolution to assist in the planning of future trials.

**Figure 2:**
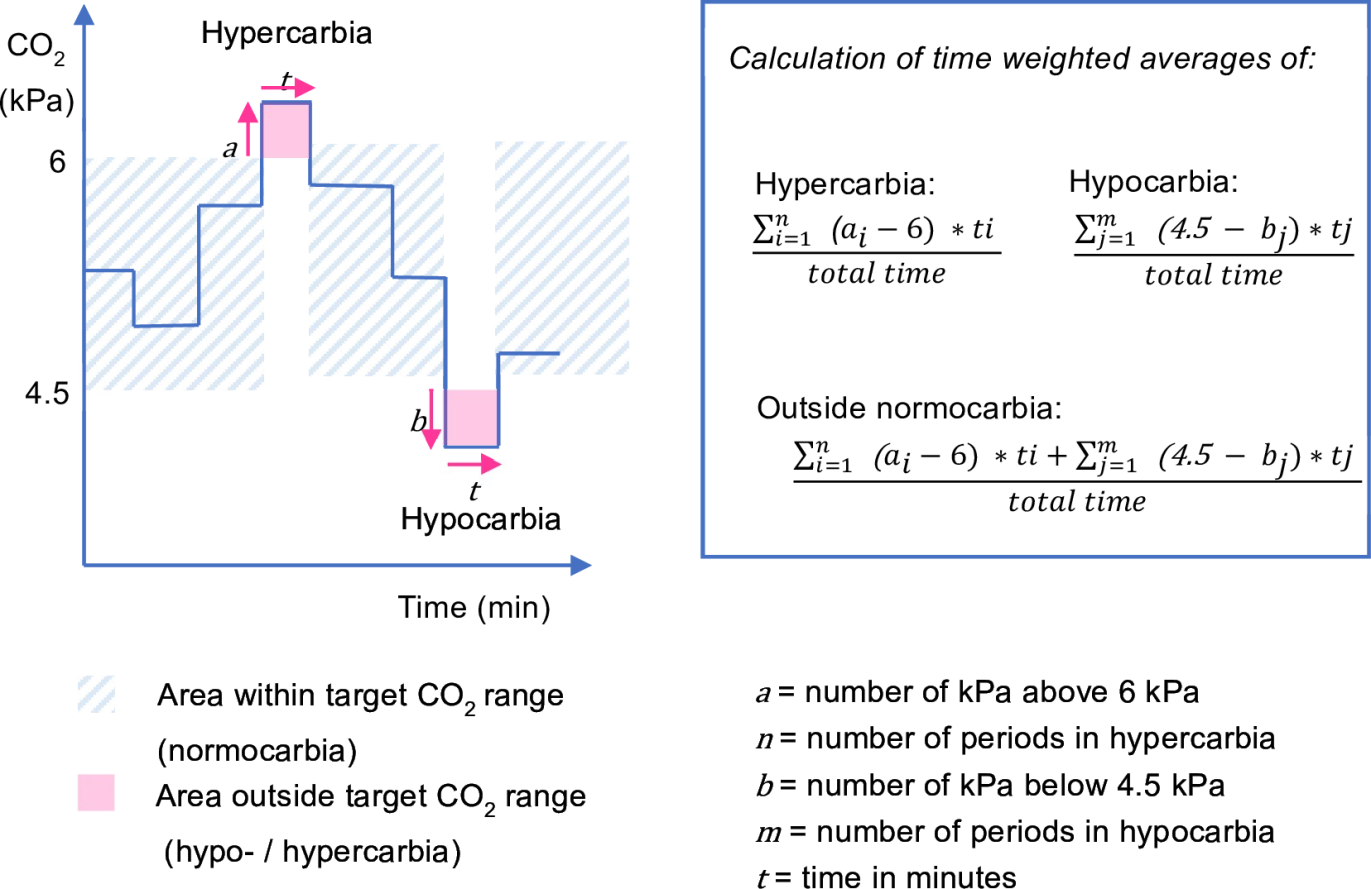
Graphical display and formulas used to calculate time-weighted averages for hypocarbia, hypercarbia and overall time spent outside the CO_2_ target range. The formulas account for both the severity of the hypocarbia/hypercarbia and time spent under hypocarbia/above hypercarbia in the numerator, divided by the total time until censoring for each patient. CO_2_, carbon dioxide.

- The *primary feasibility outcome* is adherence to the assigned ventilation mode among enrolled patients, measured from randomisation to censoring (48 hours after randomisation, extubation, discharge from PICU, or death, whichever comes first) among enrolled patients. This is measured as the proportion of time under invasive ventilation spent in the assigned ventilation mode. Discontinuations from the assigned mode lasting 15 min or less are excluded. We set a threshold for adherence to the allocated mode of 80%, meaning that patients who are ventilated with the assigned mode during ≥80% of their time in the study are considered adherent.
- The *primary physiological outcome* is the proportion of time spent within the target CO_2_ range (normocarbia, defined as CO_2_≥35 mm Hg or 4.5 kPa and ≤45 mm Hg or 6 kPa) as measured by end-tidal CO_2_ (ETCO_2_) recorded by the ventilation device and transferred to the electronic health record system every minute from randomisation to censoring. This is calculated as the total number of minutes spent with ETCO_2_ within the target CO_2_ range divided by the total number of minutes ventilated from randomisation to censoring. Arterial blood gas analysis is the gold standard to assess ventilation; however, this is an invasive and intermittent measurement. Trends in ETCO_2_ are non-invasive, continuously available at bedside and serve as a proxy for changes in partial pressure of CO_2_ in arterial blood (PaCO_2_) in children and neonates.^15^
- *Secondary feasibility outcomes* describe recruitment and implementation aspects of the study (table 2). These include the number of patients who were screened, were missed, gave consent and were randomised and enrolled per month, reasons for protocol violations; and times from randomisation to protocol violation, from start of ventilation to screening, from start of ventilation to randomisation and from consent to randomisation. The technical feasibility of digital data extraction is assessed through the proportion of enrolled participants with complete primary and secondary outcome data extracted from the electronic patient records.
- *Secondary physiological outcomes* on hypocarbia, hypercarbia or both are calculated using time-weighted averages to account not only for the time spent outside the target CO_2_ range but also for the magnitude of the difference from the target CO_2_ range (table 2, figure 2). The oxygenation index measured using peripheral oxygen saturation (SpO_2_) and partial pressure of arterial oxygen (PaO_2_) are also secondary physiological outcomes. While ETCO_2_, transcutaneous CO_2_ and SpO_2_ are time series measurements, the PaCO_2_ and PaO_2_ are obtained from intermittent blood gas analyses. To assess the later intermittent measurements over study time, each value will be carried on until the time of the next measurement.^16^

**Table 2:** Outcome measures

Primary feasibility outcome	Adherence to the allocated ventilation mode among randomised and enrolled patients, measured from randomisation until censoring (at 48 hours since randomisation, extubation, discharger or death, whichever comes first).
Secondary feasibility outcomes	Number of patients who were screened, missed (screening failure), gave consent and were randomised/enrolled per month Reasons for protocol violations (time taken to obtain written informed consent exceeded 24 hours; written informed consent obtained but no data collected; patient randomised but did not meet study specified inclusion/exclusion criteria for enrolment in the study; patient randomised to an incorrect age group strata; patient randomised but ventilation mode received is not the same as the randomised allocation; other) Time from randomisation to protocol violation Proportion of enrolled participants with complete primary and secondary outcome data extracted from the electronic patient record Time from ventilation start until screening Time from ventilation start until randomisation Time between consent and randomisation
Primary physiological outcome	Proportion of time spent within the target CO_2_ range (normocarbia, defined as CO_2_ ≥35 mm Hg or 4.5 kPa and ≤45 mm Hg or 6 kPa) measured using end-tidal CO_2_ recorded every minute from randomisation until censoring.
Secondary physiological outcomes (figure 2)	Time-weighted average of hypocarbia (CO_2_<35 mm Hg or 4.5 kPa), from randomisation until censoring and measured using: continuous end-tidal CO_2_ intermittent arterial blood gas analysis continuous transcutaneous CO_2_ measurements calibrated with results of arterial blood gas analyses Time-weighted average of hypercarbia (CO_2_>45 mm Hg or 6 kPa), from randomisation until censoring and measured using: continuous end-tidal CO_2_ intermittent arterial blood gas analysis continuous transcutaneous CO_2_ measurements calibrated with results of arterial blood gas analyses Time-weighted average of hypocarbia and hypercarbia (ie, outside normocarbia, CO_2_<35 mm Hg or 4.5 kPa; or CO_2_>45 mm Hg or 6 kPa), from randomisation until censoring and measured using: continuous end-tidal CO_2_ intermittent arterial blood gas analysis continuous transcutaneous CO_2_ measurements calibrated with results of arterial blood gas analyses Oxygenation index, from randomisation until censoring and measured using: Peripheral oxygen saturation index (OSI): MAP * FiO_2_ * 100 / SpO_2_ (MAP=mean airway pressure, FiO_2_=fraction of inspired oxygen, SpO_2_=peripheral oxygen saturation) Arterial oxygenation index (OI): MAP * FiO_2_ * 100 / PaO_2_ (PaO_2_=partial pressure of oxygen in arterial blood)

CO_2_carbon dioxideFiO_2_fraction of inspired oxygenMAPmean airway pressurePaO_2_partial pressure of oxygen in arterial bloodSpO_2_peripheral oxygen saturation

### Sample size

We aim to recruit a total of 60 patients, 30 per study arm. To determine the feasibility to progress to a larger trial, we define feasibility of adherence to the allocated ventilation mode (primary feasibility outcome) if two-thirds or more (≥67%) of patients are ventilated with the allocated mode during ≥80% of the time from randomisation until censoring. If 47 out of 60 patients are ventilated with the allocated mode for ≥80% of the study time, this would yield a feasibility of adherence of 78% with a one-sided lower 95% CI of 68%. A sample size of 60 participants will allow us to assess other feasibility outcomes and is considered sufficient to assess the variation and distribution of continuous physiological outcomes for future trials, based on previously published flat^17^,^20^ and stepped rules of thumb for pilot trials.^21^

### Data collection

There are two primary sources of data for this study: (1) data manually entered into REDCap and (2) the electronic medical record.

Data for documentation and monitoring purposes that require manual collection is related to screening, consenting, protocol deviations, and adverse events for reporting and monitoring purposes. These data are recorded using a short electronic Case Report Form (eCRF) in REDCap electronic data capture tools hosted at the University Children’s Hospital Zurich. The data are accessible only to the study team, are password-protected and backed up nightly to the servers of the University Children’s Hospital Zurich. These data will be used to calculate a subset of the secondary feasibility outcomes.

Data related to the intervention and physiological outcomes of the trial is captured digitally through the electronic health record. This has two advantages: (1) there is almost no data entry burden for study coordinators, physicians or nurses on the care team and (2) time series data can be collected at a much higher temporal resolution than what would be possible if done manually. Electronic data sources include: clinical information system (CGM Clinical – Phoenix), intensive care clinical information system (MetaVision), laboratory information system and hospital administration system (Hospis). In MetaVision, time series parameters are collected every minute. The following medical devices feed data directly into the intensive care information system: mechanical ventilator (Bellavista) for all ventilation-related parameters and ETCO_2_, transcutaneous CO_2_ monitor (Sentec), SpO_2_ monitor (Pulsoximeter Rad-87 Massimo). The clinical data warehouse team at the University Children’s Hospital Zurich extracts information from the participants’ electronic health record from randomisation to censoring as a comma-separated value file. In addition, the mandatory dataset for the Swiss Intensive Care registry (MDSi, https://www.sgi-ssmi.ch/de/datensatz.html) is also captured in the clinical data warehouse and helps describe the patient cohort. The following information is extracted by the clinical data warehouse team to describe the study population, to calculate the primary feasibility outcome, and the primary and secondary physiological outcomes:

- Baseline characteristics, such as age, sex, height, weight, Paediatric Index of Mortality-2 (PIM-2),^22 23^ which indicates the risk of death as a percentage from 0 to 100 (the higher the percentage, the higher the risk) and is used as a proxy for overall severity of disease, main diagnosis, date and time of admission, start of ventilation and randomisation, arterial blood gas analysis, endotracheal tube size and cuff.
- Respiratory mechanics and intervention-related parameters during the study period, including PEEP, MAP, peak inspiratory pressure, respiratory rate, tidal volume, minute volume, inspiratory time, leakage percentage, fraction of inspired oxygen (FiO_2_) and SpO_2_
- Outcome-related parameters during the study period such as ventilation mode, ETCO_2_, transcutaneous CO_2_, date and time of extubation, discharge or death, inspired fraction of oxygen ratio (SpO_2_/FiO_2_), results of consecutive blood gas analyses and arterial partial pressure of oxygen and inspired fraction of oxygen ratio (PaO_2_/FiO_2_). Arterial blood gas analyses are planned to be obtained every 6 hours during the study period. If the arterial line is removed during the study period, blood gas analyses may be obtained preferably from a capillary sample.

To test the electronic patient data extraction procedures, we performed test extractions of the above parameters using retrospective data from the electronic health records of 60 patients with approved general consent. This allowed us to streamline data extraction and cleaning procedures before recruitment started. In addition, we will perform a manual data verification to validate the data extraction after enrolment of the first 2 to 10 patients.

### Statistical analysis plan

The primary analysis will be performed on an intention-to-treat cohort. In case patients participate in the trial multiple times, the unit of analysis will shift from individual patients to individual admissions. This distinction will be appropriately handled through multilevel analyses. Results will be presented as effect estimates and 95% CIs, without the inclusion of p values. While missing data imputation will not be performed, outliers management in time series data will be addressed through the application of kernel smoothing.^24^

We will use descriptive statistics to summarise patient characteristics, adverse events and the distribution of feasibility and physiological outcomes. Categorical variables will be described using frequencies and percentages, continuous variables following a normal distribution using mean and SD and non-normally distributed variables using median and IQR. Normality will be assessed graphically with plots (histograms and Q–Q plots).

The analysis of both primary and secondary feasibility outcomes will be descriptive.

To analyse the primary physiological outcome, we will calculate the mean difference in the proportion of time spent in normocarbia (outcome) between the two ventilation modes (explanatory variable) using a linear mixed-effects regression model if the outcome is normally distributed. The stratification variable (age group) will be included as a fixed effect, and admissions will be nested within patients to account for correlated observations from patients potentially included more than once in the study. If the outcome has a skewed distribution, we will use quantile regression, also adjusting for stratification. The study is not powered to detect differences in physiological outcomes between the two ventilation models. Secondary physiological outcomes will be calculated using time-weighted averages as described in figure 2.

For physiological outcomes, we will conduct a per-protocol analysis including patients with adhere to the assigned ventilation mode for 80% or more of the study time.

As sensitivity analysis, we will calculate physiological outcomes using individual target CO_2_ ranges manually entered into the electronic information system by prescribing physicians when available, instead of standard normocarbia ranges. We will also assess the robustness of the results by testing different kernel sizes for smoothing and also present crude (not smoothed) results.

Secondary analyses will explore triggers and factors associated with deviations from the assigned ventilation mode (if patients received a mechanical ventilation mode different from that assigned at randomisation). We will examine interactions with age for physiological outcomes using plots and likelihood ratio tests. We will assess agreement between PaCO_2_ and both ETCO_2_ and transcutaneous CO_2_ values around the time windows when blood gas analysis was performed.

Further details on the statistical analyses can be found in the online supplemental material (statistical analysis plan).

### Ethics and dissemination

This study received its first ethical approval from the Cantonal Ethics Commission of Zurich on 6 February 2023 (Business Administration System for Ethics Committees ‘BASEC’ identification number 2022–00829). Substantial changes to the study setup and study organisation, the protocol and relevant study documents will be submitted to the ethics committee for approval before implementation. Written informed consent is obtained from parents or guardians or adolescents aged 14 years and older (online supplemental material file, information and consent form for parents). Trial and participant data are only accessible to authorised personnel who need the data to fulfil their duties within the scope of the study. Participants are coded with a unique participant identifier on the CRFs and other study-specific documents. Coding documents linking the participant’s personal information to unique study identifiers are kept in a password-protected Excel spreadsheet with limited access.

Study results will be disseminated using traditional (submission for publication in a peer-reviewed journal and presentations at conferences) and novel (social media, web or podcasts) formats. Information about the study is available on the PICU page of the University Children’s Hospital Zurich—Children’s Research Centre webpage (https://www.kispi.uzh.ch/forschungszentrum/forschungsgebiete/intensivmedizin-neonatologie-fzk). Participants are asked during the consent process if they would like to be informed of the results of this study and, if so, their email address is collected for the purpose of sharing relevant publications.

### Patient and public involvement

Patient and public involvement has not been implemented in this pilot study, which focuses on feasibility and mechanistic aspects rather than translation to clinical care at this stage. We fully recognise the importance of involving patient families in the early stages of the research plan. If this pilot trial is successful, we plan to involve patient representatives in the design of future larger follow-up trials, with a stronger focus on patient-centred outcomes and clinical implications.

### Monitoring and safety

Monitoring is performed according to the predefined monitoring plan of the Children’s Research Center of the University Children’s Hospital Zurich. The site initiation visit took place prior to the enrolment of any participants. There are three routine onsite monitoring visits (first as soon as possible after the enrolment of the first two participants, second after enrolment of 30 participants, third and close-out visit after enrolment of the last participant or in case of premature closure). The monitor reviews all informed consent forms and at least one complete review of the trial master file/investigator site file. Eligibility, primary endpoint, serious adverse events and protocol-specific safety parameters are reviewed for the first two trial participants and up to 20% of trial participants enrolled at the time of the visit, if available. Randomisation and allocation will be reviewed for at least 20% of trial participants.

The study design does not pose any serious additional risks to patients, as patients require the use of invasive mechanical ventilation due to their clinical situation, and both modes of invasive mechanical ventilation are part of accepted standard of care at the study site. Exemptions from expedited reporting are allowed when the serious adverse event is either a clear result of the underlying condition or a known complication of the condition. Because both interventions are currently used in paediatric critical clinical care, the following serious adverse events that may occur in mechanically ventilated critically ill patients are exempt from expedited reporting: pneumothorax, endotracheal tube obstruction, pneumonia, sepsis, cerebral oedema, seizures, stroke, intraventricular haemorrhage, cardiac failure, atelectasis, air leaks, pulmonary haemorrhage, subglottic stenosis, vocal cord injury and need for extracorporeal membrane oxygenation. Deaths cases will be reported. We will submit an annual safety report to the ethics committee. Because this is a pilot feasibility trial investigating two currently accepted and commonly used ventilation modes with a high anticipated safety profile and minimal associated risk, with a main focus on digital trial data extraction procedures, a formal data safety monitoring board was not considered necessary.

## Trial status and timeline

The recruitment period started on 15 January 2024. The first patient was enrolled on 25 January 2024. This protocol manuscript was submitted on 29 March 2024. At the time of submission, we had enrolled 21 participants. The last enrolment occurred during the peer-review process, on 18 September 2024; however, no modifications were made to the protocol during that time.

## Significance

The richness and high resolution of data routinely collected in intensive care provides a unique opportunity to study the efficacy of different procedures and treatments on physiological outcomes in children and can enable more efficient digital trial designs leveraging data extraction procedures to reduce workload associated with trials. This study serves as proof of concept for future larger digital clinical trials with patient-centred outcomes using electronic health record data. These data can assist in the design of future trials by providing necessary information for sample size calculation and by identifying potential difficulties and solutions.

## supplementary material

10.1136/bmjopen-2024-087043 online supplemental file 1

10.1136/bmjopen-2024-087043 online supplemental file 2

10.1136/bmjopen-2024-087043 online supplemental file 3
