# Supplementary material for: Comparison of carbon dioxide control during pressure controlled versus pressure-regulated volume controlled ventilation in children (CoCO2): protocol for a pilot digital randomised controlled trial in a quaternary paediatric intensive care unit
Source: BMJ Open. 2025 Jan 11;15(1):e087043. doi: 10.1136/bmjopen-2024-087043 (PMC11752026; doi:10.1136/bmjopen-2024-087043)
Supplement: online supplemental file 1 [file bmjopen-15-1-s001.pdf]

Liebe Eltern

Wir möchten Sie hiermit über unsere klinische Studie informieren. Ihr Kind ist minderjährig und kann nicht selbständig in das geplante Forschungsvorhaben einwilligen. Wir lassen Ihnen daher diese Informationsschrift zukommen mit der Bitte, die Zustimmung zur Teilnahme Ihres Kindes an der Studie zu prüfen. Sie als Eltern können dann stellvertretend einwilligen.

Eventuell wurde Ihr Kind im Rahmen einer Notfallbehandlung bereits in dieser klinische Studie eingeschlossen, und wir wenden uns nun an Sie, um Ihre Erlaubnis zu erhalten, Ihr Kind in der Studie weiter zu betreuen und Informationen für die Studie zu sammeln. Es gibt zwei Gründe, warum wir zum Zeitpunkt der invasiven mechanischen Beatmung Ihres Kindes nicht an Sie herangetreten sind: Erstens glauben wir, dass dies eine sehr stressige Zeit für die Eltern ist, um sich für die Teilnahme an einer Studie zu entscheiden. Ebenso wollten wir nicht die Notfallbetreuung Ihres Kindes verzögern. Zweitens, die beiden invasiven Beatmungsformen, die wir in dieser Studie vergleichen, werden bereits auf pädiatrischen Intensivstationen im Rahmen der Standardversorgung verwendet.

Im Folgenden wird Ihnen die klinische Studie dargestellt: zunächst in einer Zusammenfassung, damit Sie wissen, um was es geht.

UNIVERSITÄTS-
KINDERSPITAL
ZÜRICH

Forschungszentrum
für das Kind FZK

Anfrage zur Teilnahme an medizinischer Forschung:

Vergleich des Gasaustauschs (CO2) bei zwei invasiven mechanischen Beatmungsformen: eine digitale klinische Studie

Liebe Eltern

Wir fragen Sie hier an, ob Sie bereit wären, die Zustimmung zur Teilnahme ihres Kindes (der Patientin/des Patienten) an der Studie zu geben.

Die Teilnahme ist freiwillig. Alle Daten, die in diesem Projekt erhoben werden, unterliegen strengen Datenschutzvorschriften.

Das Forschungsvorhaben wird durchgeführt von Dr. med. Rebeca Mozun am Universitäts-Kinderspital Zürich. Bei Interesse informieren wir Sie gerne über die Ergebnisse aus dem Forschungsvorhaben.

In einem Gespräch erklären wir Ihnen die wichtigsten Punkte und beantworten Ihre Fragen. Damit Sie sich ein Bild machen können, hier das Wichtigste. Im Anschluss folgen dann weitere, detaillierte Informationen.

Warum führen wir dieses Forschungsvorhaben durch?

- Viele Kinder, die auf pädiatrischen Intensivstationen Atemunterstützung durch invasive mechanische Beatmung benötigen, werden mit dem sogenannten klassischen PC-Modus (PC = pressure controlled, druck-kontrolliert) beatmet, um einen ausreichenden Gasaustausch zu erreichen. Im klassischen Modus stellt der/die Arzt/Ärztin den Luftdruck ein, der in die Lunge gelangen soll.
- In unserem Forschungsvorhaben wollen wir herausfinden, ob eine andere auch häufig verwendete invasive mechanische Beatmungseinstellung, der adaptive (angepassten) PRVC-Modus, besser zur Kontrolle des Gasaustauschs (ausgeatmetes Kohlendioxid, CO_2) ist als der klassische PC-Modus. Im adaptiven Modus legt der/die Arzt/Ärztin ein Zielmenge an Luft fest, das in die Lunge gelangen soll. Ein Algorithmus liefert den Druck und misst die vorherigen Atemzügen, um das Zielmenge zu erreichen.
- Weiter wollen wir diese Studie als Konzeptnachweis für eine „digitale Studie“ verwenden, indem wir klinische und Beatmungsdaten von PatientInnen direkt aus elektronischen Patientenakten und Überwachungssystemen digital entnehmen und analysieren.

Was muss die Patientin/der Patient bei einer Teilnahme tun? – Was geschieht bei einer Teilnahme?

- Wenn Ihr Kind teilnimmt, wird sie/er zufällig in zwei Gruppen eingeteilt. Ihr Kind wird mit dem klassischen Beatmungsmodus oder dem angepassten Beatmungsmodus behandelt.
- Wir werden klinische und Beatmungsdaten bis zum 2. Tag der invasiven mechanischen Beatmung seit Sudienbeginn oder bis Ihr Kind nicht mehr auf eine Beatmung angewiesen ist.
- Während der 48 Stunden der Studie werden alle 6 Stunden kleine Blutproben entnommen, um die Blutgase zu analysieren. Kinder mit Beatmung haben dafür häufig Blutentnahmen, welche über eine bereits liegende Leitung/Infusion entnommen werden. Ihr Kind wird dabei nur in Ausnahmefällen, wenn keine Leitung mehr vorhanden ist, gepiekst.
- Für Ihr Kind werden keine weiteren Untersuchungen für die Studie neben der Routineversorgung anfallen.

Welcher Nutzen und welches Risiko sind damit verbunden?

Nutzen

- Ihr Kind hat keinen direkten Nutzen bei der Teilnahme an diesem Forschungsvorhaben.
- Die Studienteilnahme hilft künftigen Patientinnen und Patienten, die mit invasiver mechanischer Beatmung behandelt werden müssen. Es wird auch helfen, zukünftige grössere Studien mit patientenrelevanten Ergebnissen zu planen, in denen Daten von Beatmungsgeräten und klinischen Routinedaten digital erfasst werden können.

Risiko und Belastung

- Diese Studie birgt keine zusätzlichen Risiken für Ihr Kind. Die in dieser Studie verglichenen Beatmungsmethoden werden in der klinischen Routinepraxis auf pädiatrischen Intensivstationen bereits häufig eingesetzt.

Mit Ihrer Unterschrift am Ende des Dokuments bezeugen Sie, dass Sie die Zustimmung zur Teilnahme Ihres Kindes an der Studie freiwillig geben und dass Sie die Inhalte des gesamten Dokuments verstanden haben.

Detaillierte Information

1. Ziel und Auswahl

Unser Forschungsvorhaben bezeichnen wir in dieser Informationsschrift als *Studie*. Wenn Sie als Angehörige zur Teilnahme Ihres Kindes an der Studie einwilligen, ist sie/er eine *Studienteilnehmerin/*ein *Studienteilnehmer.*

Ihr Kind hat Atembeschwerden und benötigt die Unterstützung eines Beatmungsgerätes, damit dem Körper genügend Sauerstoff zugeführt werden kann. Dazu wurde Ihrem Kind ein Atemschlauch (Tubus) in die Luftröhre eingelegt, um ihm das Atmen zu erleichtern. Dieser Schlauch wird an ein sogenanntes Beatmungsgerät angeschlossen, das Luft und Sauerstoff in die Lungen Ihres Kindes drückt, um ihm beim Atmen zu helfen. Beatmungsgeräte bieten mehrere Beatmungsformen an. Es ist jedoch noch unklar, welche Form bei Kindern sich am besten eignet.

In dieser Studie werden wir die TeilnehmerInnen zufällig (randomisiert) in eine der beiden Gruppen einteilen. Im klassischen PC („pressure controlled“)-Modus stellt der Arzt den Luftdruck ein, der in die Lunge gelangen soll, während das Beatmungsgerät die Luftmenge misst, das in die Lunge gelangt. Im adaptiven PRVC („pressure regulated volume control“)-Modus legt der Arzt eine Zielmenge fest, das in die Lunge gelangen soll. Ein Algorithmus liefert den Druck (mit einem ähnlichen Fluss wie im „PC“-Modus) und misst die Lungenelastizität (Dehnbarkeit) in den vorherigen Atemzügen, um das Zielvolumen zu erreichen.

Wir wollen mit dieser Studie untersuchen, ob ein adaptiver PRVC-Beatmungsmodus besser als ein klassischer PC-Beatmungsmodus zur Kontrolle des Gasaustauschs (ausgeatmetes Kohlendioxid, CO_2) ist.

Wir fragen Sie als Eltern an, da alle Personen teilnehmen können, die
- jünger als 18 Jahre alt sind und mehr als 2 kg wiegen,
- während ihres Aufenthalts auf der pädiatrischen Intensivstation eine invasive mechanische Beatmung zur Unterstützung der Atmung benötigen,
- keine der folgenden Krankheiten haben: zyanotische Shuntläsion, pulmonale Hypertonie, intrakranielle Hypertonie.
- einen arteriellen Zugang benötigen

2. Allgemeine Informationen

Wir wissen noch wenig darüber, welches die beste Art der invasiven mechanischen Beatmung bei Kindern ist. Zurzeit basiert deswegen die Wahl der Beatmungsmethode bei Kindern meist auf früheren Erfahrungen und der Präferenz des behandelnden Arztes.

Wir möchten daher herausfinden, ob eine von zwei häufig verwendeten invasiven mechanischen Beatmungsmodus, adaptive PRVC-Modus und klassisch PC-Modus, besser zur Kontrolle des Gasaustauschs (ausgeatmetes Kohlendioxid, CO_2) dient.

Dies ist eine randomisierte, kontrollierte Studie mit zwei invasiven mechanischen Beatmungsformen als Interventionsgruppen. Randomisiert bedeutet, dass die Patienten zufällig in eine der beiden Gruppen gewählt werden (siehe Glossar, Punkt 15).

Es ist eine monozentrische nationale Studie, sie wird nur am Kinderspital Zürich durchgeführt..

Die Beatmungsgeräte erfassen automatisch jede Minute die Daten der Beatmungsparameter und des ausgeatmeten CO_2. Diese Beatmungsparameter und die klinischen Daten, die routinemässig auf der pädiatrischen Intensivstation erfasst werden, sind durch die elektronischen Dokumentationssysteme digital verfügbar.

Wir werden klinische und Beatmungsdaten bis zum 2. Tag der invasiven mechanischen Beatmung seit Studienbeginn oder bis zur Ende der invasiven mechanischen Beatmung oder Entlassung erfassen. Während des Studienzeitraums werden wir alle 6 Stunden die Blutgase analysieren. Das Blut wird aus bereits für die klinische Versorgung platzierten Kathetern entnommen. Nur wenn diese Katheter entfernt werden, werden wir Ihr Kind pieksen, um eine kleine Probe zu entnehmen. Für diese Studie werden keine Blut- oder biologischen Proben aufbewahrt.

Wir planen, insgesamt 60 PatientInnen einzuschliessen.

Wir machen diese Studie so, wie es die Gesetze in der Schweiz vorschreiben. Ausserdem beachten wir alle international anerkannten Richtlinien. Die zuständige Ethikkommission hat die Studie geprüft und bewilligt.
Eine Beschreibung dieser Studie finden Sie auch auf der Internetseite des Bundesamtes für Gesundheit unter www.kofam.ch.

3. Ablauf

Bevor Ihr Kind in die Studie eingeschlossen wird, prüft das Studienteam, ob alle Kriterien erfüllt werden. Sie als Eltern werden in aller Ruhe und in einem guten Moment über das Projekt informiert.
Wenn möglich sprechen wir mit Ihnen als Eltern vor dem Einsatz der mechanischen Beatmung und bitten um Ihre Einwilligung für den Einschluss in diese Studie. Wir wissen, dass die Situation für Sie sehr stressig ist und Sie womöglich in Sorge um Ihr Kind kein Ohr für unsere Bitte haben. In diesem Fall dürfen wir, dies ist von der Ethikkommission so genehmigt, Ihr Kind in die Studie einschliessen und Sie hinterher informieren. Ein unabhängiger Arzt, der mit der Studie nichts zu tun hat, wird anwesend sein und schriftlich bestätigen, dass die Interessen Ihres Kindes gewahrt werden und dessen Sicherheit gewährleistet ist. Sie haben dann immer noch die Möglichkeit zu sagen, dass die Daten des Kindes nicht verwendet werden dürfen.
Wenn Ihr Kind an der Studie teilnehmen kann, wird zufällig ermittelt, ob es eine von zwei möglichen Beatmungsformen erhält. Die Patienten werden nur Untersuchungen oder Behandlungen erhalten, die für die klinische Standardversorgung erforderlich sind. Die für diese Studie verwendeten Daten werden routinemässig manuell von Ärzten und Pflegepersonal oder automatisch von Beatmungs-, Überwachungs- und Blutanalysegeräten erfasst. Wir werden diese Daten entnehmen und analysieren.

Die Hausärztin/der Hausarzt des Patienten wird über die Studienteilnahme informiert.

4. Nutzen

Ihr Kind wird persönlich keinen Nutzen von der Teilnahme an der Studie haben.
Die Resultate können wichtig sein für andere Kinder, die in der Zukunft eine invasive mechanische Beatmung brauchen. Die Resultate können auch helfen, zukünftige grössere Studien mit patientenrelevanten Ergebnissen zu planen, in denen Daten von Beatmungsgeräten und klinische Routinedaten digital erfasst werden.

5. Freiwilligkeit und Pflichten

Die Teilnahme an der Studie ist freiwillig. Wenn Ihr Kind nicht mitmacht oder Sie als Eltern später die Teilnahme zurückziehen wollen, muss dies nicht begründet werden. Die medizinische Behandlung/Betreuung ist unabhängig von diesem Entscheid gewährleistet.

Als teilnehmende Person ist es notwendig, dass

- Die Patientin/der Patient sich an die notwendigen Vorgaben und Anforderungen der Studie durch den Prüfplan hält.

- Die Prüfärztin/der Prüfarzt über den Verlauf der Erkrankung informiert wird und neue Symptome, neue Beschwerden und Änderungen im Befinden gemeldet werden (auch nach Studienende/-abbruch, z.B. bis die unerwünschte Wirkung abklingt);
- Die Prüfärztin/der Prüfarzt über die gleichzeitige Behandlung und Therapie bei anderen Ärztinnen oder Ärzten und über die Einnahme von Medikamenten informiert wird.

6. Risiken und Belastungen für die Teilnehmenden

Diese Risiken können bei beiden Beatmungsformen auftreten: beatmungsinduzierte Lungenverletzungen, beatmungsbedingte Ereignisse wie Pneumonie und Nebenwirkungen der Sedierung wie Neurotoxizität und die Belastung der Familie durch die Behinderung von Interaktionen. Die Risiken sind somit nicht studienspezifisch, da Ihr Kind als Standardbehandlung eine invasive Atemunterstützung bekommt.

7. Alternative

Die Teilnahme an der Studie ist mit Chancen und Risiken verbunden. Die Notwendigkeit einer mechanischen Beatmung beruht jedoch auf einer klinischen Entscheidung des Arztes. Wenn Sie nicht an dieser Studie teilnehmen möchten, wählt der/die Arzt/Ärztin einen invasiven mechanischen Beatmungsmodus nach sein/seine persönlicher Präferenz aus. Ihre Prüfärztin/Ihr Prüfarzt wird Sie im Gespräch hierzu beraten.

8. Ergebnisse aus der Studie

Es gibt
1. individuelle Ergebnisse der Studie, die den Patient direkt betreffen,
2. objektive End-Ergebnisse der gesamten Studie.

Zu 1: Die Prüfärztin/der Prüfarzt wird Sie als Eltern stellvertretend im Verlauf der Studie über alle für den Teilnehmenden persönlich wichtigen, neuen Ergebnisse und Erkenntnisse informieren. Sie werden mündlich und schriftlich informiert und können dann erneut entscheiden, ob die Patientin/der Patient an der Studie weiter teilnehmen soll. Sie können auch auf diese Informationen verzichten, dazu müssen Sie sich mit einer Person aus dem Studienteam in Verbindung setzen.
Zu 2: Die Prüfärztin/der Prüfarzt der Studie kann Ihnen am Ende der Studie eine Zusammenfassung der Gesamtergebnisse zukommen lassen.

9. Vertraulichkeit der Daten und Proben

9.1. Datenverarbeitung von Verschlüsselung

Für diese Studie werden medizinische Daten Ihres Kindes erfasst und bearbeitet, teilweise in automatisierter Form. Bei der Datenerhebung werden die Daten unverschlüsselt. Unverschlüsselt bedeutet, dass die Daten und Proben identifiziert werden können. In dieser Studie werden Bezugsdaten, die Ihr Kind identifizieren könnten (z.B. der Name Geburtsdatum etc.), gelöscht und durch einen Code ersetzt werden. Wir werden jedoch das Alter des Kindes in Tagen angeben. Die genaue Erfassung des Alters ist wichtig, weil das Alter einen grossen Einfluss auf die Entwicklung und Funktion der Lunge bei Kindern hat. Die Schlüssel-Liste bleibt immer am Universitäts-Kinderspital Zürich.

Nur sehr wenige Fachpersonen werden die unverschlüsselten Daten des Patienten sehen und zwar nur, um Aufgaben im Rahmen der Studie zu erfüllen. Diese Personen unterliegen der Schweigepflicht. Sie als Angehörige haben stellvertretend das Recht auf Einsicht in die Daten der Patientin/des Patienten.

9.2. Datenschutz und Schutz der Proben

Manchmal gibt es die Vorgabe bei einer Publikation, dass Einzel-Daten (sogenannte Roh-Daten) übermittelt werden müssen. Wenn Einzel-Daten übermittelt werden, dann sind die Daten immer verschlüsselt und somit ebenfalls nicht zu Ihrem Kind rückverfolgbar. Alle Vorgaben des Datenschutzes werden eingehalten und wir werden den Namen Ihres Kindes weder in einer Publikation noch im Internet öffentlich machen. Diese Daten können verschlüsselt im Rahmen dieser Studie in eine andere Datenbank versendet werden oder für zukünftige, noch nicht näher bezeichnete Forschungsprojekte verwendet werden. Der Datenschutz hat dabei stets oberste Priorität.
Der Sponsor ist dafür verantwortlich zu sorgen, dass im Ausland die gleichen Standards wie in der Schweiz eingehalten werden.

9.3. Einsichtsrechte bei Kontrollen

Möglicherweise wird diese Studie durch die zuständige Ethikkommission oder durch die Institution, die die Studie veranlasst hat, vor Ort überprüft. Der Prüfarzt muss für solche Kontrollen die persönlichen und medizinischen Daten offenlegen. Ebenso kann es sein, dass bei Schäden auch ein Vertreter der Versicherung diese Daten ansehen muss. Alle Personen müssen absolute Vertraulichkeit wahren.
Es ist möglich, dass der nachbehandelnde Arzt kontaktiert wird, um Auskunft über den Gesundheitszustand im Rahmen der Studie zu geben.

10. Rücktritt

Ihr Kind kann jederzeit von der Studie zurücktreten und die Teilnahme beenden, wenn sie/er das wünscht oder Sie als Angehörige das entscheiden. Die bis dahin erhobenen Daten und Proben werden in diesem Fall allerdings noch verschlüsselt ausgewertet.
Nach der Auswertung werden die Daten anonymisiert. Die Schlüsselzuordnung wird vernichtet, so dass danach niemand mehr erfahren kann, vom wem die Daten ursprünglich stammten. Dies dient vorrangig dem Datenschutz.

11. Entschädigung

Wenn Ihr Kind bei dieser Studie teilnimmt, bekommt sie/er dafür keine Entschädigung.

12. Haftung

Das Universitäts-Kinderspital Zürich (der Sponsor), die die Studie veranlasst hat und für die Durchführung verantwortlich ist, haftet für Schäden, die der Patientin/dem Patienten im Zusammenhang mit der Forschungshandlungen entstehen könnten. Die Voraussetzungen und das Vorgehen dazu sind gesetzlich geregelt.
Obwohl diese Studie kein vorhersehbares Risiko beinhaltet, haftet das Kinderspital Zürich nach den gesetzlichen Bestimmungen für alle Schäden, die im Rahmen dieser Studie entstehen könnten.
Sollte die Patientin/der Patient durch die Teilnahme an dieser Studie einen Schaden erleiden, so wenden Sie sich bitte an die Prüfärztin/den Prüfarzt.

13. Finanzierung der Studie

Die Studie wird vollständig von einem Finanzierungszuschuss des Forschungszentrum für das Kind, Kinderspital Zürich bezahlt.

14. Kontaktperson(en)

Sie dürfen jederzeit Fragen zur Studien stellen. Auch bei Unsicherheiten oder Notfällen, die während der Studie oder danach auftreten, wenden Sie sich bitte an:

Name: Dr. med. Rebeca Mozun, PhD
Abteilung: Postdoktorandin, Abteilung für Intensivmedizin und Neonatologie

UNIVERSITÄTS-
KINDERSPITAL
ZÜRICH
Forschungszentrum
für das Kind FZK

Adresse:	Universitäts-Kinderspital Zürich –Eleonorenstiftung<br>Steinwiesstrasse 75, CH-8032 Zürich
Telefon:	044 266 84 83
Telefonnummer für Notfälle (24 h):	044 266 71 11
Email:	research.ipsneo@kispi.uzh

15. Glossar (erklärungsbedürftige Begriffe);

- Was heisst „randomisiert"?
  Bei vielen Studien werden zwei oder mehrere unterschiedliche Arten der Behandlung verglichen. Zum Beispiel vergleicht man ein Prüfpräparat/ein echtes Medikament mit einem Placebo. Man bildet dann zwei Gruppen von Teilnehmenden. Die einen bekommen das Prüfpräparat/das echte Medikament und die anderen das Placebo. „Randomisieren" bedeutet dann, dass ausgelost wird, wer in welche Gruppe kommt. Es ist bei einem solchen Test also Zufall, ob man das Prüfpräparat/das echte Medikament erhält oder das Placebo.

- „Sponsor": Der Sponsor ist eine Person oder Institution mit Sitz oder Vertretung in der Schweiz, die für die Veranlassung einer Studie, namentlich für dessen Einleitung, Management und Finanzierung in der Schweiz die Verantwortung übernimmt.

- PC („pressure controlled")-Modus: PC-Modus ist eine Methode zur Beatmung von Kindern mit einem mechanischen Beatmungsgerät, wo der Arzt den Luftdruck einstellet, der in die Lunge gelangen soll, während das Beatmungsgerät die Luftmenge misst, das in die Lunge gelangt.

- PRVC („pressure regulated volume control")-Modus: PRVC ist eine Methode zur Beatmung von Kindern mit einem mechanischen Beatmungsgerät, wo der Arzt eine Zielmenge festlegt, das in die Lunge gelangen soll. Ein Algorithmus liefert den Druck (mit einem ähnlichen Fluss wie im „PC"-Modus) und misst die Lungenelastizität (Dehnbarkeit) in den vorherigen Atemzügen, um das Zielvolumen zu erreichen.

UNIVERSITÄTS-
KINDERSPITAL
ZÜRICH

Forschungszentrum für das Kind FZK

Einwilligungserklärung

Schriftliche Einwilligungserklärung zur Teilnahme an einer klinischen Studie
Bitte lesen Sie dieses Formular sorgfältig durch. Bitte fragen Sie, wenn Sie etwas nicht verstehen oder wissen möchten. Für die Teilnahme der Patientin/des Patienten ist Ihre schriftliche Einwilligung notwendig.

**BASEC-Nummer (nach Einreichung):**	2022-00829
**Titel der Studie (wissenschaftlich und Laiensprache)**:	“Comparison of carbon dioxide control during pressure controlled (PC) versus pressure regulated volume control (PRVC) ventilation in children (CoCO$_2$): a digital, randomized controlled trial”<br><br>**Vergleich des Gasaustauschs (CO$_2$) bei zwei invasiven mechanischen Beatmungsformen: eine digitale klinische Studie**
**Verantwortliche Institution (Sponsor mit Adresse)**:	Universitäts-<br>Kinderspital Zürich –Eleonorenstiftung<br>Dr. med. Rebeca Mozun, PhD<br>Postdoktorandin, Abteilung für Intensivmedizin und Neonatologie<br>Steinwiesstrasse 75<br>CH-8032 Zurich
**Ort der Durchführung**:	Universitäts-<br>Kinderspital Zürich –Eleonorenstiftung
**Prüfärztin/Prüfarzt am Studienort:**<br>Name und Vorname in Druckbuchstaben:	
**Teilnehmerin/Teilnehmer:**<br>Name und Vorname in Druckbuchstaben:<br>Geburtsdatum:	

- Ich wurde als Eltern des Studienteilnehmenden (oben benannt) von der unterzeichnenden Prüfärztin/vom unterzeichnenden Prüfarzt mündlich und schriftlich über den Zweck und den Ablauf der Studie mit invasiven mechanischen Beatmungsmethoden über mögliche Vor- und Nachteile sowie über eventuelle Risiken informiert.
- Ich bestätige, dass ich im Sinne der Person entscheide, nämlich, dass mein Kind an der Studie teilnimmt. Stellvertretend akzeptiere ich die mündliche und schriftliche Information. Ich hatte genügend Zeit, diese Entscheidung zu treffen.
- Die Fragen im Zusammenhang mit der Teilnahme an dieser Studie sind mir beantwortet worden. Ich behalte die schriftliche Information und erhalte eine Kopie der schriftlichen Einwilligungserklärung.
- Ich bin einverstanden, dass die zuständigen Fachleute des Sponsors und der zuständigen Ethikkommission zu Prüf- und Kontrollzwecken in die unverschlüsselten Daten der Patientin/des Patienten Einsicht nehmen dürfen, jedoch unter strikter Einhaltung der Vertraulichkeit.

- Bei Ergebnissen, die direkt die Gesundheit der Patientin/des Patienten betreffen, werde ich stellvertretend informiert. Wenn ich denke, dass dies nicht in ihrem/seinem Sinne ist, informiere ich die Prüfärztin/den Prüfarzt.
- Ich weiss, dass die persönlichen Daten nur in verschlüsselter Form zu Forschungszwecken für diese Studie weitergegeben werden können (auch ins Ausland). Die Projektleitung gewährleistet, dass der Datenschutz nach Schweizer Standard eingehalten wird.
- Ich kann stellvertretend für die Patientin/den Patient jederzeit und ohne Angabe von Gründen von der Studienteilnahme zurücktreten. Die weitere medizinische Behandlung der Patientin/des Patienten ist unabhängig von der Studienteilnahme gewährleistet. Die bis zum Rücktritt erhobenen Daten und Proben werden noch im Rahmen der Studie ausgewertet.
- Die Haftpflichtversicherung des Kinderspitals Zürich kommt für allfällige Schäden auf.

Bestätigung der Eltern: Hiermit bestätige ich/ bestätigen wir, dass das Aufklärungsgespräch stattgefunden hat und dass das urteilsunfähige Kind/ die urteilsunfähige Person in die Studienteilnahme eingewilligt hat und/oder sich keine Anzeichen von Widerstand gegen die Teilnahme erkennen lassen.

Ort, Datum, Zeit	Name und Vorname in Druckbuchstaben<br><br>Angabe der Beziehung zur Patientin/zum Patienten (Sohn/Tochter):<br><br>Unterschrift Angehörige/gesetzliche Vertretung/Eltern

Möchten Sie über Studienresultate informiert werden? _ ja _ nein

Wenn ja, bitte gebe hier Deine Email-Adresse an ……………………………………………

Bestätigung der Prüfärztin/des Prüfarztes/der Prüfperson: Hiermit bestätige ich, dass ich der oben unterzeichnenden Person in Stellvertretung der Teilnehmerin/des Teilnehmers Wesen, Bedeutung und Tragweite der klinischen Studie erläutert habe. Ich versichere, alle im Zusammenhang mit der Studie stehenden Verpflichtungen gemäss in der Schweiz geltenden Rechts zu erfüllen. Sollte ich zu irgendeinem Zeitpunkt während der Durchführung der Studie von Aspekten erfahren, welche die Bereitschaft der Teilnehmerin/des Teilnehmers an der Studie beeinflussen könnten, werde ich die Person stellvertretend umgehend darüber informieren.

Ort, Datum, Zeit	Name und Vorname der Prüfärztin/der Prüfarztes in Druckbuchstaben<br><br>Unterschrift der Prüfärztin/des Prüfarztes
