# Supplementary material for: Comparison of carbon dioxide control during pressure controlled versus pressure-regulated volume controlled ventilation in children (CoCO2): protocol for a pilot digital randomised controlled trial in a quaternary paediatric intensive care unit
Source: BMJ Open. 2025 Jan 11;15(1):e087043. doi: 10.1136/bmjopen-2024-087043 (PMC11752026; doi:10.1136/bmjopen-2024-087043)
Supplement: online supplemental file 3 [file bmjopen-15-1-s003.pdf]

Dear parents

We would like to inform you about our clinical study. Your child is a minor and cannot independently consent to the planned research project. We are therefore sending you this information document with the request that you evaluate your child's consent to participate in the study. You as parents can then give your consent on behalf of your child.

Your child may have already been included in this clinical trial as part of emergency care and we are now contacting you to obtain your permission to continue to care for your child in the trial and to collect information for the trial. There are two reasons why we did not approach you at the beginning your child's invasive mechanical ventilation: Firstly, we believe that this is a very stressful time for parents to decide to participate in a study. Similarly, we did not want to delay your child's emergency care. Secondly, the two forms of invasive ventilation we are comparing in this study are already used in paediatric intensive care units as part of standard care.

The following is a summary of the clinical trial so that you know what it is about.

Request to participate in medical research:

Comparison of gas exchange (CO2) in two invasive mechanical ventilation modes: a digital clinical study

Dear parents

We are asking you here whether you would be willing to give your consent for your child (the patient) to participate in the study.

Participation is voluntary. All data collected in this project is subject to strict data protection regulations.

The research project is being conducted by Dr Rebeca Mozun at the University Children's Hospital Zurich. If you are interested, we will be happy to inform you about the results of the research project.

We will explain the most important points to you and answer your questions. To give you an idea, here are the most important points. Further detailed information will then follow.

Why are we carrying out this research project?

- Many children who require respiratory support through invasive mechanical ventilation in paediatric intensive care units are ventilated using the so-called classic PC mode (PC = pressure controlled) in order to achieve sufficient gas exchange. In classic mode, the doctor sets the air pressure that is to enter the lungs.
- In our research project, we want to find out whether another commonly used invasive mechanical ventilation setting, the adaptive (customised) PRVC mode, is better for controlling gas exchange (exhaled carbon dioxide, CO_2) than the classic PC mode. In adaptive mode, the doctor sets a target amount of air that should enter the lungs. An algorithm provides the pressure and measures the previous breaths to reach the target amount.
- We also want to use this study as proof of concept for a "digital study" by digitally extracting and analysing clinical and ventilation data from patients directly from electronic patient records and monitoring systems.

What does the patient have to do when participating? - What happens if the patient participates?

- If your child takes part, he/she will be randomly assigned into one of two groups. Your child will be treated with the classic ventilation mode or the adaptive ventilation mode.
- We will keep clinical and ventilation data until the 2nd day of invasive mechanical ventilation since the start of the study or until your child is no longer dependent on ventilation.
- During the 48 hours of the study, small blood samples are taken every 6 hours to analyse the blood gases. Children on mechanical ventilation often have blood

samples taken via an existing line/infusion. Your child will only be pricked in exceptional cases if there is no longer a line available.
- Your child will not have any further examinations for the study apart from routine care.

What benefits and risks are associated with this?

Benefit

- Your child will not benefit directly from participating in this research project.
- Participation in the study will help future patients who need to be treated with invasive mechanical ventilation. It will also help to plan future larger studies with patient-relevant outcomes in which data from ventilators and routine clinical data can be recorded digitally.

Risk and exposure

- This study does not harbour any additional risks for your child. The ventilation methods compared in this study are already frequently used in routine clinical practice in paediatric intensive care units.

With your signature at the end of the document, you attest that you voluntarily give your consent to your child's participation in the study and that you have understood the contents of the entire document.

Detailed information

1. Goal and selection

In this information document, we refer to our research project as a *study*. If you, as a relative, consent to your child's participation in the study, then he/she are a study *participant.*

Your child has breathing difficulties and requires the support of a ventilator so that sufficient oxygen can be supplied to the body. A breathing tube has been inserted into your child's throat to make breathing easier. This tube is connected to a ventilator, which forces air and oxygen into your child's lungs to help them breathe. Ventilators offer several forms of ventilation. However, it is still unclear which form is most suitable for children.

In this study, we will randomise the participants into one of the two groups. In the classic PC ("pressure controlled") mode, the physician sets the air pressure that should enter the lungs, while the ventilator measures the amount of air that enters the lungs. In adaptive PRVC ("pressure regulated volume control") mode, the doctor sets a target volume to be delivered to the lungs. An algorithm delivers the pressure (with a similar flow as in "PC" mode) and measures the lung elasticity (distensibility) in the previous breaths to reach the target volume.

With this study, we want to investigate whether an adaptive PRVC ventilation mode is better than a classic PC ventilation mode for controlling gas exchange (exhaled carbon dioxide, CO_2).

We ask you as parents, as all persons can participate who

- are younger than 18 years old and weigh more than 2 kg,
- require invasive mechanical ventilation to support breathing during their stay in the paediatric intensive care unit,
- have none of the following diseases: cyanotic shunt lesion, pulmonary hypertension, intracranial hypertension.
- require an arterial access

2. General information

We still know little about the best type of invasive mechanical ventilation for children. At present, the choice of ventilation method in children is therefore mostly based on previous experience and the preference of the treating physician.

We would therefore like to find out whether one of two commonly used invasive mechanical ventilation modes, adaptive PRVC mode and classic PC mode, is better for controlling gas exchange (exhaled carbon dioxide, CO_2).

This is a randomised controlled study with two invasive mechanical ventilation forms as intervention groups. Randomised means that the patients are assigned by chance to one of the two groups (see glossary, point 15).

It is a monocentric national study and is only being conducted at the Children's Hospital Zurich.

The ventilators automatically record data on ventilation parameters and exhaled CO_2 every minute. These ventilation parameters and the clinical data that are routinely recorded in the paediatric intensive care unit are available digitally via the electronic documentation systems.
We will collect clinical and ventilation data until day 2 of invasive mechanical ventilation from the start of the study or until the end of invasive mechanical ventilation or discharge. During the study period, we will analyse blood gases every 6 hours. Blood will be drawn from catheters already in place for clinical care. Only when these catheters are removed will we prick your child to take a small sample. No blood or biological samples will be stored for this study.

We plan to enrol a total of 60 patients.

We conduct this study in accordance with Swiss law. We also observe all internationally recognised guidelines. The responsible ethics committee has reviewed and authorised the study. A description of this study can also be found on the website of the Federal Office of Public Health at www.kofam.ch.

3. Procedure

Before your child is included in the study, the study team will check whether all criteria are met. As parents, you will be informed about the project at a good time.
If possible, we will speak to you as parents before mechanical ventilation is used and ask for your consent to be included in this study. We know that the situation is very stressful for you and that you may not be able to listen to our request because you are worried about your child. In this case, we are authorised by the ethics committee to include your child in the study and inform you afterwards. An independent doctor who has nothing to do with the study will be present and will confirm in writing that your child's interests are being safeguarded and that his or her safety is guaranteed. You then still have the option of saying that your child's data may not be used.
If your child is eligible to participate in the study, he/she will be randomised to receive one of two possible forms of ventilation. Patients will only receive investigations or treatments that are required for standard clinical care. The data used for this study will be routinely collected manually by doctors and nurses or automatically by ventilation, monitoring and blood analysers. We will collect and analyse this data.

The patient's general practitioner will be informed about participation in the study.

4. Benefit

Your child will not personally benefit from participating in the study.
The results may be important for other children who need invasive mechanical ventilation in the future. The results may also help to plan future larger studies with patient-relevant outcomes in which data from ventilators and routine clinical data are recorded digitally.

5. Voluntariness and obligations

Participation in the study is voluntary. If your child does not take part or you as parents later wish to withdraw participation, there is no need to give reasons for this. Medical treatment/support is guaranteed regardless of this decision.

As a participating person, it is necessary that

- The patient adheres to the necessary specifications and requirements of the study through the protocol.
- The investigator is informed about the course of the disease and new symptoms, new complaints and changes in well-being are reported (even after the end of the study/cancellation, e.g. until the adverse effect subsides);
- The investigator is informed about concurrent treatment and therapy by other physicians and about the intake of medication.

6. Risks and burdens for the participants

These risks can occur with both forms of ventilation: ventilation-induced lung injuries, ventilation-related events such as pneumonia and side effects of sedation such as neurotoxicity and the burden on the family due to the obstruction of interactions. The risks are therefore not specific to the study, as your child will receive invasive respiratory support as standard treatment.

7. Alternative

Participation in the study is associated with opportunities and risks. However, the need for mechanical ventilation is based on a clinical decision by the doctor. If you do not wish to participate in this study, the doctor will choose an invasive mechanical ventilation mode according to his/her personal preference. Your investigator will advise you on this during the interview.

8. Results from the study

There are

1. individual results of the study that directly affect the patient,
2. objective final results of the entire study.

Re 1: The investigator will inform you as parents on behalf of the patient during the course of the study about all new results and findings that are personally important for the participant. You will be informed verbally and in writing and can then decide again whether the patient should continue to participate in the study. You can also choose not to receive this information, in which case you must contact a member of the study team.
Re 2: The investigator of the study can send you a summary of the overall results at the end of the study.

9. Confidentiality of data and samples

9.1. Data processing of encryption

Your child's medical data will be collected and processed for this study, partly in automated form. During data collection, the data will be unencrypted. Unencrypted means that the data and samples can be identified. In this study, reference data that could identify your child (e.g. name, date of birth, etc.) will be deleted and replaced by a code. However, we will record the child's age in days. It is important to record the exact age because age has a major influence on the development and function of the lungs in children. The key list always remains at the University Children's Hospital Zurich.

Only very few professionals will see the patient's unencrypted data, and only to fulfil tasks within the scope of the study. These persons are subject to a duty of confidentiality. As relatives, you have the right to view the patient's data on behalf of the patient.

9.2. Data protection and protection of samples

Sometimes there is a requirement in a publication that individual data (so-called raw data) must be transmitted. If individual data is transmitted, the data is always encrypted and therefore cannot be traced back to your child. All data protection regulations are complied with and we will not publicise your child's name in a publication or on the Internet. This data may be encrypted and sent to another database as part of this study or used for future, as yet unspecified research projects. Data protection will always have top priority.
The sponsor is responsible for ensuring that the same standards are maintained abroad as in Switzerland.

9.3. Inspection rights during inspections

This study may be audited by the responsible ethics committee or by the institution, who initiated the study on site. For such checks, the investigator must disclose personal and medical data. It is also possible that in the event of damage a representative of the insurance company must view this data. All persons must maintain absolute confidentiality.
It is possible that the doctor providing follow-up treatment will be contacted to obtain information about the health status as part of the study.

10. Withdrawal
Your child can withdraw from the study at any time and end participation if he/she wishes or if you as a relative decide to do so. In this case, however, the data and samples collected up to that point will still be analysed in encrypted form.
After evaluation, the data is anonymised. The key assignment is destroyed so that nobody can find out who originally provided the data. This is primarily for data protection purposes.

11. Compensation
If your child takes part in this study, he/she will not receive any compensation.

12. Liability
The University Children's Hospital Zurich (the sponsor), which initiated the study and is responsible for its implementation, is liable for any damage that the patient may suffer in connection with the research activities. The requirements and procedure for this are regulated by law.
Although this study does not involve any foreseeable risk, the Children's Hospital Zurich is liable in accordance with the statutory provisions for any damage that may arise in the context of this study. If the patient suffers any harm as a result of participating in this study, please contact the investigator.

13. Financing the study
The study is fully funded by a grant from the Children's Research Centre of the University Children's Hospital Zurich.

14. Contact person(s)
You may ask questions about the studies at any time. Please also contact us if you have any uncertainties or emergencies that arise during or after the study:

Name: Dr Rebeca Mozun, PhD
Department: Postdoctoral researcher, Department of Intensive Care Medicine and Neonatology
University Children's Hospital Zurich -Eleonore Foundation
Address: Steinwiesstrasse 75, CH-8032 Zurich

Phone: 044 266 84 83
Telephone number for emergencies (24 h): 044 266 71 11

Email: research.ipsneo@kispi.uzh

15. Glossary (terms requiring explanation);

- What does "randomised" mean?
  Many studies compare two or more different types of treatment. For example, an investigational product/real medication is compared with a placebo. Two groups of participants are then formed. One group receives the investigational product/the real drug and the other the placebo. "Randomising" then means drawing lots to determine who goes into which group. In such a test, it is therefore a matter of chance whether you receive the investigational product/the real drug or the placebo.

- "Sponsor": The sponsor is a person or institution domiciled or represented in Switzerland that assumes responsibility for the initiation of a study, in particular for its initiation, management and financing in Switzerland.

- PC ("pressure controlled") mode: PC mode is a method of ventilating children with a mechanical ventilator, where the doctor sets the air pressure to enter the lungs while the ventilator measures the amount of air entering the lungs.

- PRVC ("pressure regulated volume control") mode: PRVC is a method of ventilating children with a mechanical ventilator where the doctor sets a target volume to be delivered to the lungs. An algorithm delivers the pressure (with a similar flow as in "PC" mode) and measures the lung elasticity (distensibility) in the previous breaths to reach the target volume.

UNIVERSITÄTS-
KINDERSPITAL
ZÜRICH

Forschungszentrum
für das Kind FZK

Declaration of consent

Written declaration of consent to participate in a clinical trial
Please read this form carefully. Please ask if there is anything you do not understand or would like to know. Your written consent is required for the patient's participation.

**BASEC number (after submission):**	2022-00829
**Title of the study**<br>**(scientific and lay language)**:	"Comparison of carbon dioxide control during pressure controlled (PC) versus pressure regulated volume control (PRVC) ventilation in children (CoCO_2): a digital, randomized controlled trial"<br><br>**Comparison of gas exchange (CO_2) with two invasive mechanical ventilation modes: a digital clinical study**
**Responsible institution**<br>**(sponsor with address)**:	University<br>Children's Hospital Zurich -Eleonore Foundation<br>Dr Rebeca Mozun, PhD<br>Postdoctoral researcher, Department of Intensive Care Medicine and Neonatology<br>Steinwiesstrasse 75<br>CH-8032 Zurich
**Place of realisation**:	University<br>Children's Hospital Zurich -Eleonore Foundation
**Investigator at the place of study:**<br>Surname and first name in block capitals:	
**Participant:**<br>Surname and first name in block capitals:<br>Date of birth:	

- As the parent of the study participant (named above), I have been informed verbally and in writing by the undersigned investigator about the purpose and procedure of the study with invasive mechanical ventilation methods, about possible advantages and disadvantages as well as about possible risks.
- I confirm that I am making a decision in the person's best interests, namely that my child takes part in the study. I accept the verbal and written information on his/her behalf. I have had sufficient time to make this decision.
- The questions related with participation in this study have been answered. I will keep the written information and receive a copy of the written declaration of consent.
- I agree that the responsible experts of the sponsor and the responsible ethics committee may inspect the patient's unencrypted data for testing and monitoring purposes, but under strict confidentiality.
- In the case of results that directly affect the patient's health, I will be informed on behalf of the patient. If I think that this is not in his/her best interest, I will inform the investigator.

- I am aware that the personal data can only be passed on in encrypted form for research purposes for this study (including abroad). The project management guarantees that data protection in accordance with Swiss standards will be observed.
- I can withdraw from participation in the study on behalf of the patient at any time and without giving reasons. The patient's continued medical treatment is guaranteed regardless of participation in the study. The data and samples collected up to the time of withdrawal will still be analysed as part of the study.
- The liability insurance of the Children's Hospital Zurich covers any damage.

Confirmation of the parents: I/we hereby confirm that the informed consent discussion has taken place and that the child/person lacking capacity has consented to participate in the study and/or that there are no signs of resistance to participation.

Place, date, time	Surname and first name in block capitals<br><br>Indicate the relationship to the patient (son/daughter):<br><br>Signature of relatives/legal representative/parents

Would you like to be informed about study results? Yes no

If yes, please enter your email address here ....

Confirmation of the investigator/research coordinator: I hereby confirm that I have explained the nature, significance and scope of the clinical trial to the person signing above on behalf of the participant. I confirm that I will fulfil all obligations in connection with the study in accordance with Swiss law. If at any time during the conduct of the study I become aware of aspects that could influence the participant's willingness to take part in the study, I will inform the person immediately.

Place, date, time	Surname and first name of the investigator in block capitals<br><br>Signature of the investigator
