# Supplementary material for: Comparison of carbon dioxide control during pressure controlled versus pressure-regulated volume controlled ventilation in children (CoCO2): protocol for a pilot digital randomised controlled trial in a quaternary paediatric intensive care unit
Source: BMJ Open. 2025 Jan 11;15(1):e087043. doi: 10.1136/bmjopen-2024-087043 (PMC11752026; doi:10.1136/bmjopen-2024-087043)
Supplement: Supplementary file 4 [file bmjopen-2024-087043.reviewer_comments.pdf]

PEER REVIEW HISTORY

BMJ Open publishes all reviews undertaken for accepted manuscripts. Reviewers are asked to complete a checklist review form and are provided with free text boxes to elaborate on their assessment. These free text comments are reproduced below.

ARTICLE DETAILS

Title (Provisional)

Comparison of carbon dioxide control during pressure controlled versus pressure regulated volume controlled ventilation in children (CoCO2): protocol for a pilot digital randomized controlled trial in a quaternary paediatric intensive care unit

Authors

Mozun, Rebeca; Chopard, Daphné; Zapf, Florian; Baumann, Philipp; Brotschi, Barbara; Adam, Anika; Jaeggi, Vera; Bangerter, Beat; Gibbons, Kristen S; Burren, Juerg; Schlapbach, Luregn J

VERSION 1 - REVIEW

**Reviewer**	**1**
**Name**	**Luo, Mengqiang**
**Affiliation**	**Huashan Hospital Fudan University**
**Date**	**01-Jun-2024**
**COI**	**None competing interests.**

Overall, the design of this clinical randomized trial is relatively reasonable. However, my main concern remains the relatively small sample size, with only 30 participants per study arm, despite the authors addressing this limitation by calculating the sample size during the experiment design phase.

My second major concern is that the trial aims to enroll mechanically ventilated children younger than 18 years, who are eligible for randomization if their weight is above 2 kg. As we know, children's lung compliance and tolerance to hypoxia and hypercapnia change significantly as they develop. Therefore, the differences between children of different ages, such as newborns and ten-year-olds, are very pronounced. Additionally, the relatively small sample size may significantly impact the reliability of the results. It is suggested, therefore, to focus on specific age groups of children, such as those under 1 year old.

Finally, the primary feasibility outcome is measured from randomization to censoring (48 hours after randomization, extubation, discharge from PICU, or death, whichever comes first) among enrolled patients. Would it be more meaningful to have a longer follow-up period?

**Reviewer**	**2**
**Name**	**Yang, Yi**
**Affiliation**	**Southeast University Zhongda Hospital**
**Date**	**02-Nov-2024**

COI **None**

Thanks for giving me this chance to review this protocol. This is a well written, comprehensive protocol for a single-centre, open-labelled, randomized controlled pilot trial in a PICU at the University Children's Hospital Zurich to assess the feasibility of a digital PICU trial comparing two modes of invasive mechanical ventilation using CO2 control as the outcome measure. I had a few comments and hope these comments can help improvement in the manuscript.

Major comments

1. You should demonstrate that this trial is designed following the Standard Protocol Items: Recommendations for Interventional Trials Statement (SPIRIT). I notice that although the attachment provides relevant information, this issue was not specifically highlighted in the the Methods.

2. I can not find your registration in this URL using NCT058431. Maybe you provided a wrong number in the Abstract? In the ADMINISTRATIVE INFORMATION, you give a right number (NCT05843123). Please check it. Furthermore, you need to adress this information in the Methods.

3. Did you enroll the enough numbers of patients? I suggest including the number of patients enrolled so far in your manuscript.

Minor comments

1. I suggest that you can include the schedule table.

2. Please provide the date on which you obtained ethical approval.

3. Please elaborate on how the researcher should respond if a patient wishes to withdraw from the study.

VERSION 1 - AUTHOR RESPONSE

Reviewer: 1

Dr. Mengqiang Luo, Huashan Hospital Fudan University

Comments to the Author:

1. Overall, the design of this clinical randomized trial is relatively reasonable. However, my main concern remains the relatively small sample size, with only 30 participants per study arm, despite the authors addressing this limitation by calculating the sample size during the experiment design phase.

Reply 8: Aligned with a pilot study design, we acknowledge that our sample size is relatively small. However, the primary aim of this pilot study is to evaluate feasibility and gather insights to inform the design of future larger trials, and as a pilot trial the study is thus not intended to be powered for major clinical outcomes. As a result, we do not intend to provide definitive conclusions regarding our physiological outcomes. Consistent with best practice on pilot studies, we will not report p-values in our analyses (see Methods, Statistical analysis plan, page 7). Instead, the findings will serve as a robust foundation for conducting power calculations in subsequent studies. We have now rephrased a sentence in the article summary emphasizing this.

Article summary, page 3:

> *"The outcomes of this pilot trial focus on feasibility and physiological measures, with the sample size allowing the assessment of feasibility and exploration of physiological findings to guide the design and inform power calculations of future larger trials, which should also investigate patient-centred outcomes, age-related differences, and longer follow-up time."*

2. My second major concern is that the trial aims to enrol mechanically ventilated children younger than 18 years, who are eligible for randomization if their weight is above 2 kg. As we know, children's lung compliance and

tolerance to hypoxia and hypercapnia change significantly as they develop. Therefore, the differences between children of different ages, such as newborns and ten-year-olds, are very pronounced. Additionally, the relatively small sample size may significantly impact the reliability of the results. It is suggested, therefore, to focus on specific age groups of children, such as those under 1 year old.

Reply 9: We acknowledge that the age range is broad, and agree that lung physiology changes throughout the neonatal period, infancy, childhood and adolescence. However this age range reflects the population of critically ill children exposed to mechanical ventilation, where different ventilation modes are routinely applied around the world. To mitigate potential imbalances, randomization is stratified by age groups. As outlined in the response above, in this pilot study we will not have sufficient power to detect differences in physiological outcomes across several age groups. At this stage, we are unable to modify the inclusion criteria. Nonetheless, future trials will need be powered to detect age-related differences. We believe it is important to include older children, as there is limited evidence in this age group. We now mention the relevance of investigating age-related differences when designing future larger trials in the article summary section.

Article summary, page 3:

> *"The outcomes of this pilot trial focus on feasibility and physiological measures, with the sample size allowing the assessment of feasibility and exploration of physiological findings, to guide the design and inform power calculations of future larger trials, which should also investigate patient-centred outcomes, age-related differences, and longer follow-up time."*

3. Finally, the primary feasibility outcome is measured from randomization to censoring (48 hours after randomization, extubation, discharge from PICU, or death, whichever comes first) among enrolled patients. Would it be more meaningful to have a longer follow-up period?

Reply 10: Thank you for your input. We defined the censoring period considering the high temporal resolution of the data collected. A longer follow-up period could provide additional insights and potentially reveal temporal differences depending on the number of days since the mechanical ventilation started. As the outcomes and follow-up time must be defined in advance, we are not able to revise them at this stage. However, we will take this comment into consideration when planning future larger trials.

Article summary, page 3:

> *"The outcomes of this pilot trial focus on feasibility and physiological measures, with the sample size allowing the assessment of feasibility and exploration of physiological findings, to guide the design and inform power calculations of future larger trials, which should also investigate patient-centred outcomes, age-related differences, and longer follow-up time."*

Reviewer: 2

Dr. Yi Yang, Southeast University Zhongda Hospital

Comments to the Author:

Thanks for giving me this chance to review this protocol. This is a well written, comprehensive protocol for a single-centre, open-labelled, randomized controlled pilot trial in a PICU at the University Children's Hospital Zurich to assess the feasibility of a digital PICU trial comparing two modes of invasive mechanical ventilation using CO2 control as the outcome measure. I had a few comments and hope these comments can help improvement in the manuscript.

Major comments

4. You should demonstrate that this trial is designed following the Standard Protocol Items: Recommendations for Interventional Trials Statement (SPIRIT). I notice that although the attachment provides relevant information, this issue was not specifically highlighted in the Methods.

Reply 11: Thank you for this remark. We now explicitly mention adherence to the SPIRIT statement and included its reference in the methods section.

Methods, study design and setting, page 5:

> *"We follow Standard Protocol Items for Randomized Trials (SPIRIT) reporting guidelines.[13]"*

5. I cannot find your registration in this URL using NCT058431. Maybe you provided a wrong number in the Abstract? In the ADMINISTRATIVE INFORMATION, you give a right number (NCT05843123). Please check it.

Furthermore, you need to address this information in the Methods.

Reply 12: We confirm that the clinicaltrials.gov ID number NCT05843123 is correct (https://clinicaltrials.gov/study/NCT05843123 = Study Details | Comparison of Gas Exchange Between Two Invasive Mechanical Ventilation Modes in Children | ClinicalTrials.gov).

6. Did you enrol the enough numbers of patients? I suggest including the number of patients enrolled so far in your manuscript.

Reply 13: We have updated the trial status and timeline section including information on first and last recruitment dates. Information on the recruitment rates is part of our outcomes and will be reported with the results of the study.

Trial status and timeline, page 9

> *"The recruitment period started on January, 15, 2024. The first patient was enrolled on January 25, 2024, This protocol manuscript was submitted on March 29, 2024. At time of submission we had enrolled 21 participants. The last enrolment occurred during the peer review process, on September 18, 2024; however no modifications were made to the protocol during that time."*

Minor comments

7. I suggest that you can include the schedule table.

Reply 14: We provide the schedule of trial-related activities, assessments and interventions for patient in Figure 2 (study design). We now also report in the text the planned start and end dates, as well as the actual status and timeline.

Methods, study design and setting, page 4:

> *"The study was initially planned to begin recruitment in June 2023 and expected to conclude within six months by December 2023, whereas actual recruitment started in January 2024 and was expected to be completed in July 2024. This protocol manuscript was submitted on March 29, 2024. Study recruitment was completed during the peer review process; however no changes were made to the protocol."*

Trial status and timeline, page 9

> *"The recruitment period started on January, 15, 2024. The first patient was enrolled on January 25, 2024, This protocol manuscript was submitted on March 29, 2024. At time of submission we had enrolled 21 participants. The last enrolment occurred during the peer review process, on September 18, 2024; however no modifications were made to the protocol during that time."*

Monthly recruitment rates are part of our feasibility outcomes and will be reported in the publication of study results. We therefore consider that adding an additional schedule table would not provide significant additional information.

8. Please provide the date on which you obtained ethical approval.

Reply 15: The study was first approved on 06.02.2023, and an amendment was approved on 09.05.2023. We now indicate this in the ethics and dissemination section.

Ethics and dissemination, page 8

> *"This study received first ethical approval from the Cantonal Ethics Commission of Zurich on February, 6, 2023, (Business Administration System for Ethics Committees "BASEC" identification number 2022- 00829)."*

9. Please elaborate on how the researcher should respond if a patient wishes to withdraw from the study.

Reply 16: We now included information on withdrawal procedures in the methods section.

Methods, Participants, screening and consent procedures, page 5:

> *"Withdrawal of consent does not affect subsequent medical assistance and treatment of the child. In case of withdrawal of informed consent, there is no further data collection. Data collected up to that point may be analysed in coded form and after the evaluation anonymized, or be deleted."*
